# Interleukin-1β Induces Blood–Brain Barrier Disruption by Downregulating Sonic Hedgehog in Astrocytes

**DOI:** 10.1371/journal.pone.0110024

**Published:** 2014-10-14

**Authors:** Yue Wang, Shijie Jin, Yoshifumi Sonobe, Yi Cheng, Hiroshi Horiuchi, Bijay Parajuli, Jun Kawanokuchi, Tetsuya Mizuno, Hideyuki Takeuchi, Akio Suzumura

**Affiliations:** Department of Neuroimmunology, Research Institute of Environmental Medicine, Nagoya University, Nagoya, Japan; Kyushu University, Japan

## Abstract

The blood–brain barrier (BBB) is composed of capillary endothelial cells, pericytes, and perivascular astrocytes, which regulate central nervous system homeostasis. Sonic hedgehog (SHH) released from astrocytes plays an important role in the maintenance of BBB integrity. BBB disruption and microglial activation are common pathological features of various neurologic diseases such as multiple sclerosis, Parkinson’s disease, amyotrophic lateral sclerosis, and Alzheimer’s disease. Interleukin-1β (IL-1β), a major pro-inflammatory cytokine released from activated microglia, increases BBB permeability. Here we show that IL-1β abolishes the protective effect of astrocytes on BBB integrity by suppressing astrocytic SHH production. Astrocyte conditioned media, SHH, or SHH signal agonist strengthened BBB integrity by upregulating tight junction proteins, whereas SHH signal inhibitor abrogated these effects. Moreover, IL-1β increased astrocytic production of pro-inflammatory chemokines such as CCL2, CCL20, and CXCL2, which induce immune cell migration and exacerbate BBB disruption and neuroinflammation. Our findings suggest that astrocytic SHH is a potential therapeutic target that could be used to restore disrupted BBB in patients with neurologic diseases.

## Introduction

The blood–brain barrier (BBB) is a tight seal composed of capillary endothelial cells, pericytes, and perivascular astrocytes [1]. The BBB contributes to homeostasis in the central nervous system (CNS) by limiting the entry of plasma components, erythrocytes, and immune cells from the circulating blood [2]. Astrocytes play a pivotal role in maintenance of BBB integrity via contact-dependent mechanisms and release of trophic factors [3]–[5]. In addition, a recent study revealed that Sonic hedgehog (SHH) released from astrocytes promotes BBB formation and integrity by upregulating tight junction (TJ) proteins in capillary endothelial cells [6]. Without SHH, its receptor Patched-1 (Ptch-1) suppresses a G-coupled–protein receptor Smoothened (Smo) which is critical for the activation of a transcription factor Gli-1 [7]. Gli-1 is an important regulator of TJ protein expression and BBB formation. SHH binds and inactivates Ptch-1, which allows Smo to activate Gli-1, which upregulates TJ proteins and enhances BBB integrity. Disruption of BBB integrity is frequently observed in neurologic diseases such as multiple sclerosis (MS), Parkinson’s disease, amyotrophic lateral sclerosis, and Alzheimer’s disease, suggesting that infiltrating molecules and immune cells from the blood perturb CNS homeostasis and exacerbate these disorders [8]–[13]. Microglial activation is another characteristic pathologic feature in these diseases [14]. Activated microglia release various cytotoxic factors such as nucleic acids, glutamate, reactive oxygen species (ROS), proteases, and pro-inflammatory cytokines/chemokines [15]. Interleukin-1β (IL-1β) is a major microglial pro-inflammatory cytokine that acts on both endothelial cells and astrocytes to increase BBB permeability [16]–[18]. However, the mechanisms of BBB disruption by IL-1β have not been fully elucidated. In this study, we demonstrated that IL-1β suppressed SHH expression in astrocytes and increased BBB permeability by downregulating TJ proteins in endothelial cells. Moreover, IL-1β stimulated astrocytes to secrete pro-inflammatory chemokines such as CCL2, CCL20, and CXCL2, which induce the migration of immune cells such as neutrophils, monocytes, macrophage, dendritic cells, and pathogenic T cells. Our findings reveal novel mechanisms of BBB disruption by IL-1β, and suggest that SHH could be used therapeutically against various neurologic diseases.

## Methods

### Cell cultures

Protocols for animal experiments were approved by the Animal Experiment Committee of Nagoya University (The approval number: 13122).

Mouse primary astrocyte–rich cultures were prepared from primary mixed glial-cell cultures of newborn C57BL/6J mice (SLC, Hamamatsu, Japan), as described previously [19], [20]. The purity of astrocytes was >95%, as determined by immunostaining with antibody against glial fibrillary acidic protein. Cells were cultured in maintenance medium (Dulbecco’s Modified Eagle Medium supplemented with 10% fetal bovine serum, 5 µg/ml bovine insulin, and 0.6% glucose). Astrocytes were plated at a density of 1×10^4^ cells/well in 96-well multidishes, 1×10^5^ cells/well in 24-well multidishes, or 5×10^5^ cells/well in 6-cm culture dishes. For IL-1β treatment, the cells were incubated with or without 2 ng/ml mouse recombinant IL-1β (R&D Systems, Minneapolis, MN, USA) for 24 h, and then astrocyte conditioned media (ACM) were collected and used for subsequent experiments.

The mouse brain capillary endothelial cell line, MBEC4 (a kind gift from Dr. T. Tsuruo) [21], was maintained in Dulbecco’s Modified Eagle Medium supplemented with 10% fetal bovine serum and used as an established BBB model.

### BBB permeability assay

We used MBEC4 monolayers as an *in* *vitro* BBB model, as described previously [22]. The permeability of MBEC4 monolayers was evaluated using fluorescein isothiocyanate–labeled bovine serum albumin (FITC-BSA) as a marker. Confluent monolayers of MBEC4 cells on Transwell inserts (3 µm pore size; BD Falcon, Franklin Lakes, NJ, USA) were incubated for 24 h with 2 ng/ml IL-1β, ACM, IL-1β-treated ACM, 1–100 ng/ml recombinant mouse SHH (R&D systems), 0.01–1 µM purmorphamine (a Smo agonist) (Merck Millipore, Billerica, MA, USA), or 0.3–30 µM cyclopamine (a Smo inhibitor) (Merck Millipore). Next, the monolayers were washed with assay buffer (118 mM NaCl, 4.7 mM KCl, 1.3 mM CaCl_2_, 1.2 mM MgCl_2_, 1.0 mM NaH_2_PO_4_, 25 mM NaHCO_3_, and 11 mM D-glucose, pH 7.4). This buffer (1 ml) was added to the outside of the insert (the abluminal side). Assay buffer containing 4% FITC-BSA (Sigma-Aldrich, St. Louis, MO, USA) was loaded on the luminal side of the insert and incubated for 1 h. The concentration of FITC-BSA in the abluminal chamber was determined by measuring the fluorescence (excitation, 480 nm; emission, 530 nm) using a Wallac 1420 ARVO_MX_ (PerkinElmer Japan, Yokohama, Japan). Assays were carried out in five independent trials.

### Quantitative reverse transcription-PCR

Astrocytes were collected after a 6-h incubation with 0.02–2 ng/ml IL-1β. Total RNA was extracted using the RNeasy Mini kit (Qiagen, Valencia, CA, USA) and reverse transcribed with SuperScript III (Life Technologies, Carlsbad, CA, USA) as described previously [23]. Expression levels of mRNAs encoding SHH, CXCL2, CCL2, and CCL20 were evaluated by quantitative PCR (qPCR) using TaqMan Gene Expression Master Mix (Applied Biosystems, Foster City, CA, USA) on a Rotor-Gene Q real-time PCR cycler (Qiagen). The following mouse gene–specific primers and probes were obtained from Applied Biosystems: *Shh*, Mm00436528_m1; *Cxcl2*, Mm00436450_m1; *Ccl2*, Mm00411241_m1; *Ccl20*, Mm01268754_m1; *Actb*, Mm00607939_s1; and *Gapdh*, Mm99999915_g1. Gene expression values were determined by the ΔΔC_T_ method. Levels of the mRNAs of interest were normalized to the geometric mean of *Actb* and *Gapdh* levels. Assays were carried out in five independent trials.

### Enzyme-linked immunosorbent assay (ELISA)

Astrocyte conditioned media were collected after a 24-h incubation with 0.02–2 ng/ml IL-1β, and then assessed for protein levels using ELISA kits specific for mouse SHH, CXCL2, CCL2, and CCL20 (R&D systems). Assays were carried out in five independent trials.

### Western blotting

MBEC4 cells were incubated with ACM, 2 ng/ml IL-1β, 100 ng/ml SHH, 1 µM purmorphamine, or 30 µM cyclopamine for 24 h. To assess the protein expression levels of occludin and zonula occludens-1 (ZO-1), cells were lysed in TNES buffer (50 mM Tris-HCl [pH 7.5], 150 mM NaCl, 1% Nonidet P-40, 2 mM EDTA, and 0.1% SDS) with protease inhibitor mixture (Complete Mini EDTA-free; Roche Diagnostics, Basel, Switzerland) and a phosphatase inhibitor mixture (Sigma-Aldrich) as described previously [24], [25]. Cell lysate proteins dissolved in Laemmli sample buffer (30 µg/well) were separated on 4–20% SDS-polyacrylamide gels (Mini-Protean TGX; Bio-Rad, Hercules, CA, USA) and transferred to Hybond-P polyvinylidene difluoride membranes (GE Healthcare, Piscataway, NJ, USA). The membranes were blocked for 1 h at room temperature with 5% skim milk in Tris-buffered saline containing 0.05% Tween-20, and then incubated overnight at 4°C with rabbit anti–claudin-5 monoclonal antibody (Zymed Laboratories, South San Francisco, CA, USA), rabbit anti-occludin polyclonal antibody (Zymed Laboratories), rabbit anti–ZO-1 polyclonal antibody (Zymed Laboratories), or mouse anti–β-actin monoclonal antibody (clone AC-15; Sigma-Aldrich), followed by incubation with horseradish peroxidase–conjugated secondary antibodies (GE Healthcare) for 1 h at room temperature. The signals were visualized using SuperSignal West Dura chemiluminescent substrate (Thermo Fisher Scientific, Waltham, MA, USA), and quantitated using a CS Analyzer 3.0 system (Atto, Tokyo, Japan). Assays were carried out in five independent trials.

### Statistical analysis

Statistical significance was analyzed with one-way analysis of variance followed by post-hoc Tukey’s test, using GraphPad Prism6 (GraphPad Software, La Jolla, CA, USA).

## Results

### IL-1β suppressed the protective effect of astrocytes on BBB integrity

**Figure 1:**
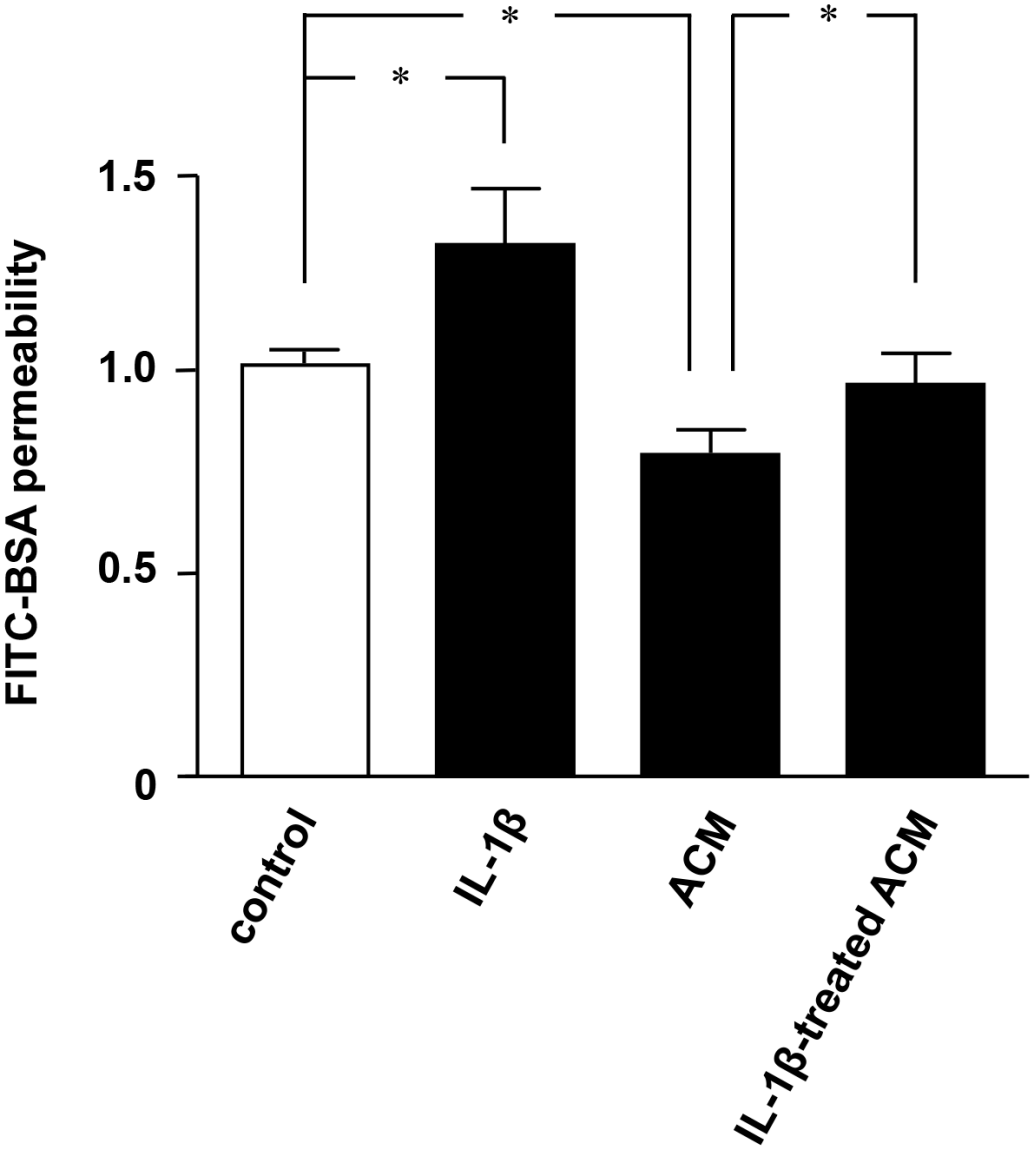
IL-1β abolishes the protective effect of astrocytes on BBB integrity. MEBC4 cells were treated for 24 h with 2 ng/ml IL-1β, ACM, or IL-1β–treated ACM for 24 h. FITC-BSA was loaded onto the luminal side of the insert for 1 h, and then the FITC-BSA levels on the abluminal side were analyzed. All quantitative data are expressed as means ± SEM (n = 5), normalized to the corresponding values from untreated cells. *, *p*<0.001.

First, we confirmed the effects of IL-1β and astrocytes on BBB integrity using MBEC4 monolayers as an *in* *vitro* BBB model. Astrocyte conditioned media (ACM) significantly decreased the permeability of BBB (Fig. 1). Treatment with IL-1β alone significantly increased the permeability of BBB, and conditioned media from IL-1β–stimulated astrocytes lost the ability to increase BBB integrity (Fig. 1). These findings suggested that IL-1β disrupts BBB integrity not only directly, but also indirectly via astrocyte dysfunction.

### IL-1β decreased astrocytic production of SHH

**Figure 2:**
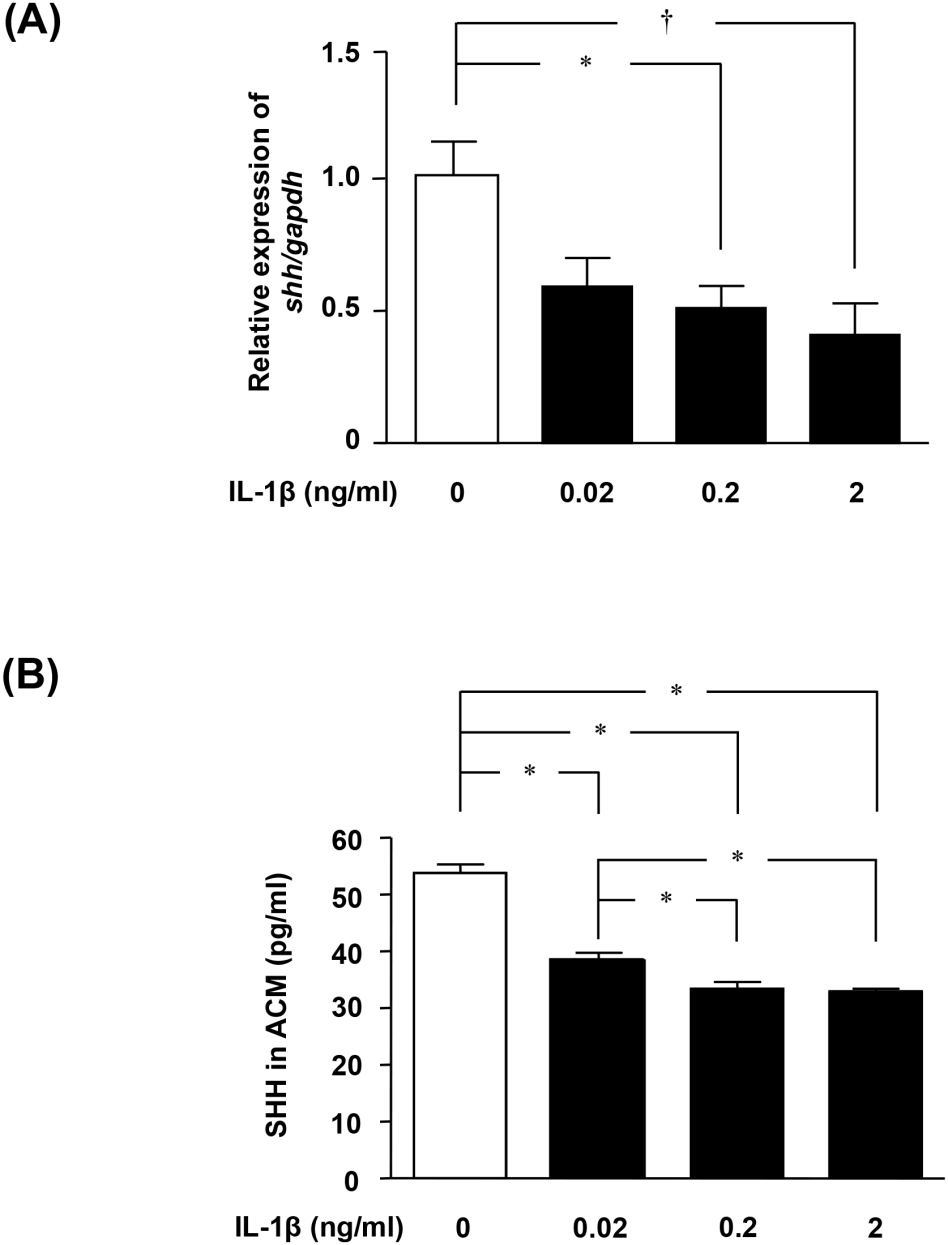
IL-1β downregulates SHH production in astrocytes. (A) *Shh* mRNA levels in astrocytes, determined by qPCR. Astrocytes were treated with IL-1β for 6 h. Values are means ± SEM (n = 5). *, *p*<0.05; †, *p*<0.01. (B) Protein levels of SHH in ACM, determined using ELISA. Astrocytes were treated with IL-1β for 24 h. Values are means ± SEM (n = 5). *, *p*<0.001.

Next, we focused on SHH, a soluble factor released from astrocytes that plays an important role in BBB maintenance. Specifically, we investigated whether IL-1β affects astrocytic SHH expression. Treatment with IL-1β significantly decreased *Shh* mRNA levels in astrocytes in a dose-dependent manner (Fig. 2A). Similar results were obtained for SHH protein levels in ACM using specific ELISA (Fig. 2B).

### SHH produced by astrocytes is critical for maintenance of BBB integrity by upregulating tight junction proteins

**Figure 3:**
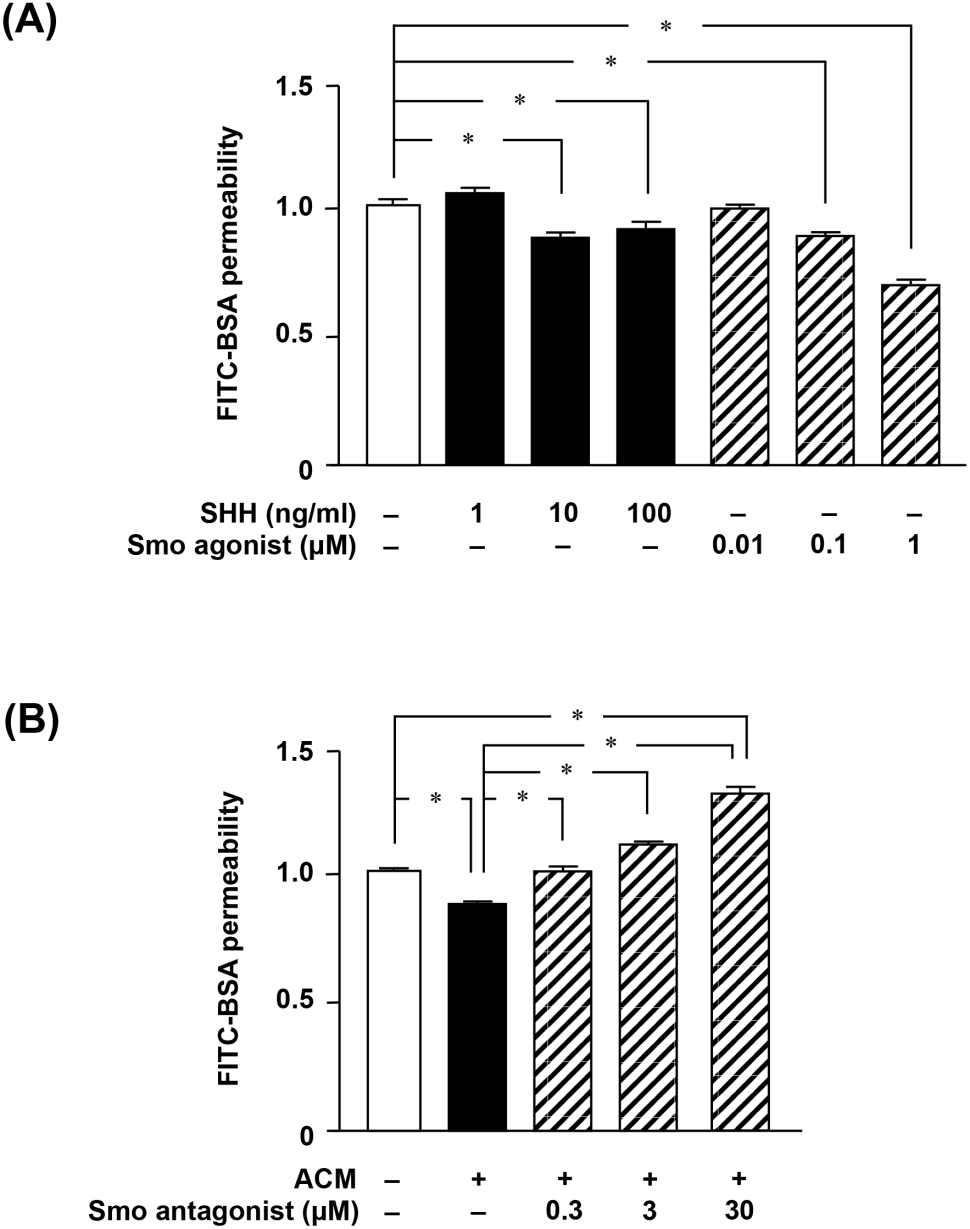
SHH signaling is critical for maintenance of BBB integrity. (A) MBEC4 cells were treated with SHH or the Smo agonist purmorphamine for 24 h. (B) MBEC4 cells were treated with ACM or the Smo antagonist cyclopamine for 24 h. FITC-BSA was loaded onto the luminal side of the insert for 1 h, and then the FITC-BSA levels on the abluminal side were analyzed. All quantitative data are expressed as means ± SEM (n = 5), normalized to the corresponding values from untreated cells. *, *p*<0.001.

**Figure 4:**
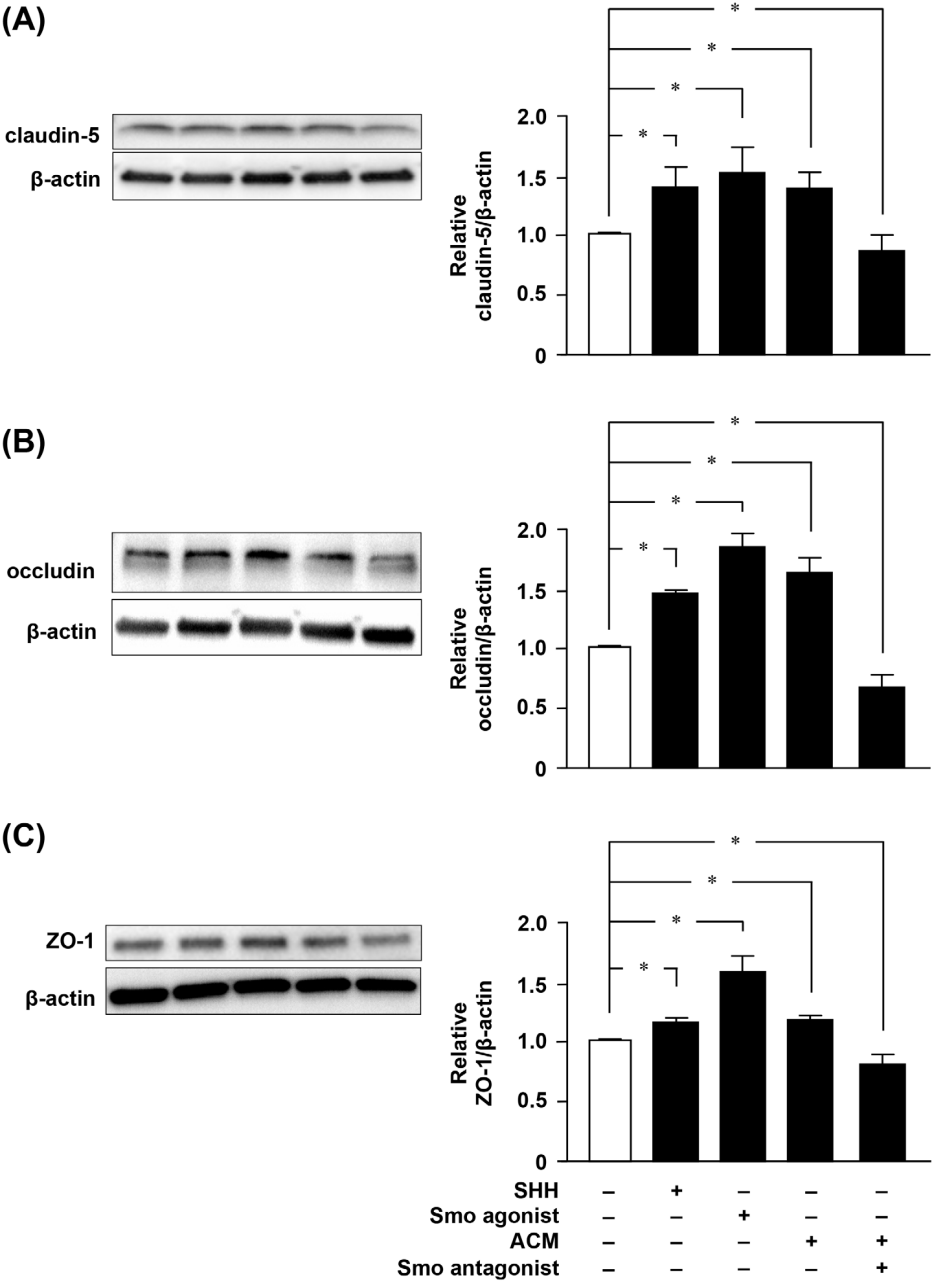
Astrocytic SHH signaling regulates expression of tight junction proteins in BBB. Western blotting of claudin-5 (A), occludin (B) and ZO-1 (C) in MBEC4 cells. Cells were treated for 24 h with ACM, SHH (100 ng/ml), the Smo agonist purmorphamine (1 µM), or the Smo antagonist cyclopamine (30 µM). All quantitative data are expressed as means ± SEM (n = 5), normalized to the corresponding values from untreated cells. *, *p*<0.05.

Next, we examined the effect of astrocytic SHH signaling on BBB function. SHH or the Smo agonist (i.e. a SHH signaling enhancer) purmorphamine significantly decreased BBB permeability (Fig. 3A). By contrast, the Smo antagonist (i.e. a SHH signaling inhibitor) cyclopamine abolished the astrocytic effect on the maintenance of BBB function (Fig. 3B). The expression levels of such TJ proteins as claudin-5, occludin, and ZO-1 were closely correlated with BBB integrity (Fig. 4A–C): levels of these proteins were highest when permeability was lowest. Activation of SHH signaling by ACM, SHH, or purmorphamine resulted in significant upregulation of these proteins, whereas the Smo antagonist cyclopamine ablated the astrocytic effect on their expression (Fig. 4A–C). These observations suggested that SHH produced by astrocytes plays a critical role in BBB integrity by upregulating expression of TJ proteins.

### IL-1β stimulated pro-inflammatory chemokine production in astrocytes

**Figure 5:**
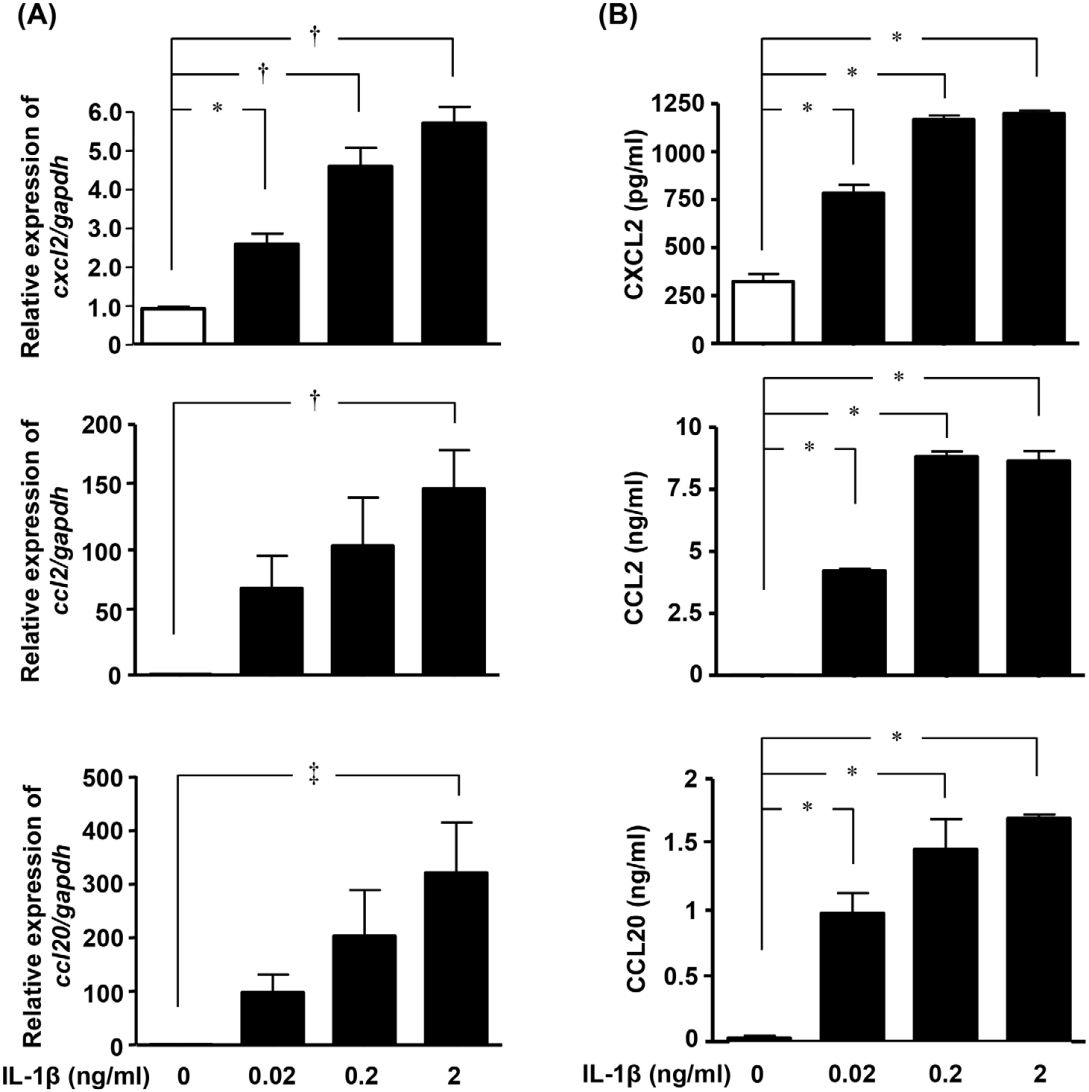
IL-1β upregulates production of pro-inflammatory chemokines in astrocytes. (A) *Cxcl2*, *Ccl2*, and *Ccl20* mRNA levels in astrocytes, determined by qPCR. Astrocytes were treated with IL-1β for 6 h. Values are means ± SEM (n = 5). *, *p*<0.05; †, *p*<0.01. (B) Protein levels of CXCL2, CCL2, and CCL20 in astrocytes, determined by ELISA. Astrocytes were treated with IL-1β for 24 h. Values are means ± SEM (n = 5). *, *p*<0.001.

Finally, we assessed the effects of IL-1β on the production of pro-inflammatory chemokines in astrocytes. Treatment with IL-1β significantly increased the mRNA and protein expression levels of CXCL2, CCL2, and CCL20 in astrocytes (Fig. 5A and 5B). These data imply that IL-1β also activates astrocytes to release these pro-inflammatory chemokines; induces migration of immune cells such as neutrophils, monocytes, macrophage, dendritic cells, and pathogenic T cells; and leads to further BBB disruption and neuroinflammation.

## Discussion

IL-1β is considered to be a critical factor for astrocyte activation in various neurologic disorders [26]. IL-1β binds to its receptor, IL-1R, whose downstream signaling activates nuclear factor-κB (NF-κB), a key player in the immune and inflammatory response in astrocytes [27], [28]. NF-κB promotes transcription of mediators of inflammation, such as pro-inflammatory cytokines/chemokines [29]. In addition, NF-κB also increases neurotrophic factor production in astrocytes [30], [31]. Therefore, IL-1β plays two opposing roles in astrocytes. Microglia are the main source of IL-1β in the CNS. A variety of stimuli, such as damage-associated molecular pattern molecules (DAMPs), amyloid β, and pro-inflammatory cytokines, trigger microglial IL-1β production via an inflammasome-dependent mechanism [32], [33]. Upregulation of IL-1β is observed in a broad spectrum of neurological diseases, including infections, trauma, stroke, and epilepsy, as well as chronic neurologic diseases such as MS, Parkinson’s disease, amyotrophic lateral sclerosis, and Alzheimer’s disease [32]; BBB disruption is associated with progression of these diseases [34]. IL-1β increases BBB permeability by downregulating TJ proteins [17], [18]. In addition, IL-1β also induces astrocytes to release vascular endothelial growth factor, which increases BBB permeability [16]. Thus, IL-1β induces BBB breakdown via both direct and indirect pathways.

**Figure 6:**
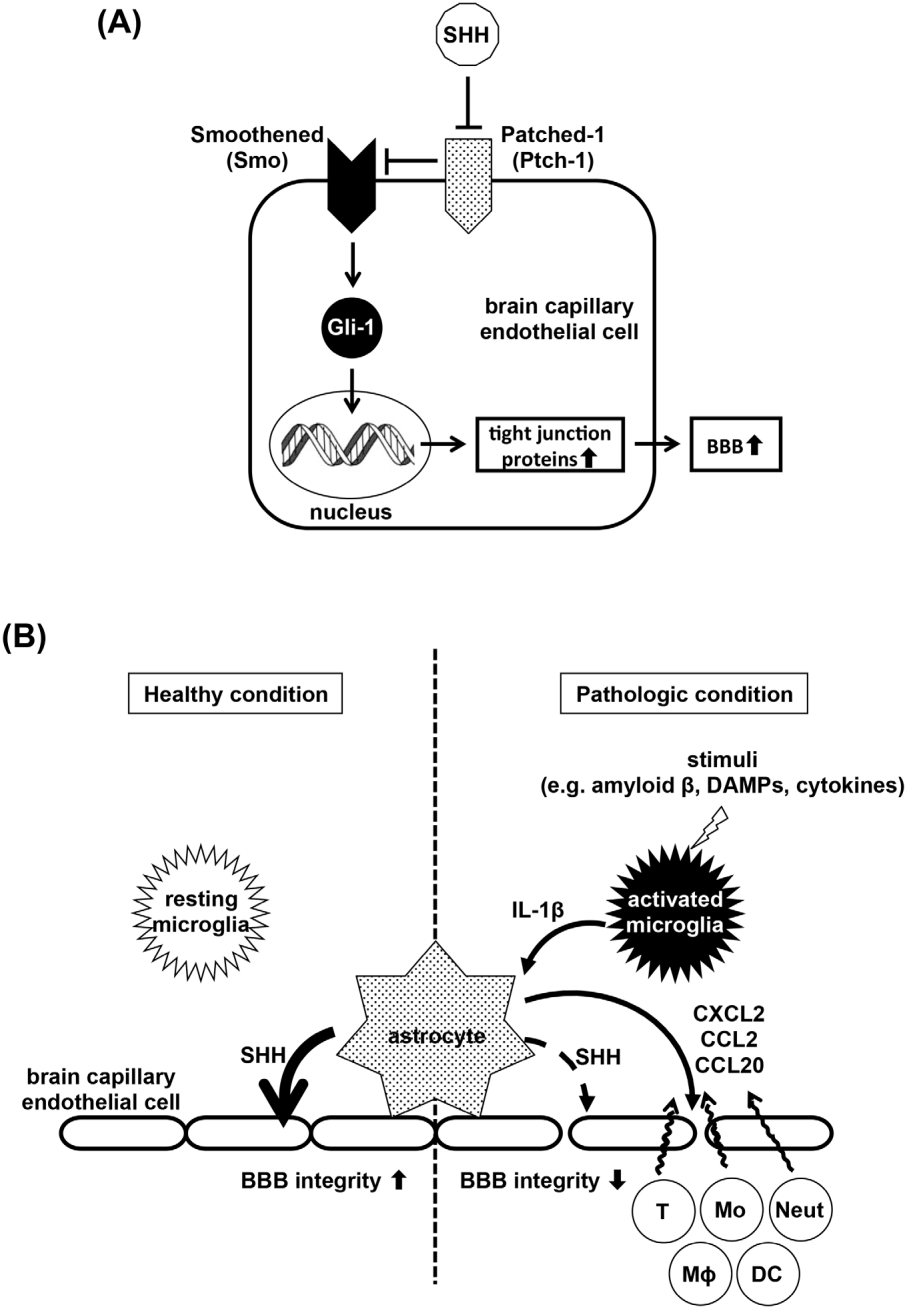
Model of the roles of the SHH and IL-1β pathways in the BBB. (A) Model of SHH signaling pathway in brain capillary endothelial cells. Secreted SHH binds and inactivates its receptor Patched-1, which allowed Smoothened to activate the transcription factor Gli-1. Gli-1 upregulates tight junction proteins and enhances BBB integrity. (B) Model of BBB breakdown by IL-1β. Under healthy conditions (left), astrocytes secrete SHH to upregulate tight junction proteins in endothelial cells and maintain BBB integrity. Under pathologic conditions (right), pathogenic stimuli such as amyloid β, DAMPs, or cytokines induce microglia to release IL-1β. IL-1β suppresses astrocytic SHH production, leading to downregulation of tight junction proteins in endothelial cells and disintegrity of the BBB. IL-1β also activates astrocytes to release pro-inflammatory chemokines such as CXCL2, CCL2, and CCL20. These chemokines induce migration of immune cells, thereby worsening BBB disruption and neuroinflammation. Neut, neutrophils; Mo, monocytes; MΦ, macrophage; DC, dendritic cells; T, T cells.

Here, we propose another novel mechanism for IL-1β–mediated BBB disruption. SHH is a critical activator of Smo–Gli-1 signaling which upregulates TJ proteins and enhances BBB integrity (Fig. 6A). A decrease in SHH allows Ptch-1 to suppress Smo–Gli-1 signaling. In the healthy state, astrocytes release SHH, which upregulates TJ proteins in endothelial cells and maintains BBB integrity (Fig. 6B, left). Once pathogenic stimuli activate microglia to release IL-1β (Fig. 6B, right), it suppresses SHH production in astrocytes, downregulates TJ proteins in endothelial cells, and disrupts BBB integrity. Moreover, IL-1β–stimulated astrocytes secrete the pro-inflammatory chemokines CXCL2, CCL2, and CCL20, which induce migration of immune cells such as neutrophils, monocytes, macrophage, dendritic cells, and pathogenic T cells. Infiltration of these cells exacerbates BBB disintegrity and subsequent neuroinflammation.

In this study, the Smo antagonist cyclopamine decreased TJ protein expression levels and BBB integrity exceeding the physiological levels (Figs. 3 and 4). Previous reports suggested that unidentified endogenous ligands of Smo seem to activate this signaling although SHH is the main regulator of Smo–Gli-1signaling [35], [36]. Our data also imply the presence of endogenous ligand(s) of Smo.

BBB disruption is a common pathologic feature of neurologic disorders such as stroke, MS, Parkinson’s disease, amyotrophic lateral sclerosis, and Alzheimer’s disease. Therefore, restoration of BBB integrity has been recognized as a therapeutic target for treatment of these diseases [8], [37]. In fact, both glucocorticoids and interferon β, both of which have been widely used for MS treatment, decrease BBB permeability [38], [39]. Moreover, the efficacy of the α4-integrin antagonist natalizumab has also demonstrated the utility of BBB-targeting drugs in treating MS [40]. By contrast, excessive immunosuppression resulting from conventional therapies for MS sometimes causes progressive multifocal leukoencephalopathy [41]. Thus, from the perspective of adverse effects, restoration of TJ proteins represents a superior therapeutic approach. Inhibition of IL-1β is a promising potential method for restoring BBB integrity [42]; however, a previous study indicated that simple blockade of IL-1β runs the risk of increasing BBB disruption, because this cytokine also enhances the protective effects of astrocytes on the BBB [26]. Treatment with SHH may circumvent this dilemma, allowing reinforcement of BBB integrity without loss of the beneficial effects of IL-1β.

During development, SHH signaling is primarily involved in CNS morphogenic events [43], whereas in adulthood, SHH participates in vascular proliferation, neurogenesis, and tissue repair in the CNS [44]. Dysregulation of SHH occurs in a variety of neurologic disorders; therefore, activation of the SHH signaling pathway, which would enhance neurogenesis and gliogenesis, has been proposed as a potential therapeutic approach for treatment of these diseases [45]. Downregulation of SHH has been observed in MS brains [46], and interferon-β treatment improves symptoms in a MS rodent model, concomitant with reduced BBB breakdown and elevated SHH expression [47]. Taken together, these observations suggest that SHH exerts a synergistic therapeutic effect by promoting CNS tissue repair while reinforcing the BBB.

This study reveals a novel mechanism for IL-1β–mediated BBB disruption: downregulation of SHH expression in astrocytes. Our findings suggest that stimulation of astrocytic SHH production could promote restoration of BBB integrity, and may therefore be useful in treating a variety of neurologic disorders.
